# Chronic mild stress alters the somatostatin receptors in the rat brain

**DOI:** 10.1007/s00213-015-4103-y

**Published:** 2015-10-14

**Authors:** A. Faron-Górecka, M. Kuśmider, M. Kolasa, D. Żurawek, K. Szafran-Pilch, P. Gruca, P. Pabian, J. Solich, M. Papp, M. Dziedzicka-Wasylewska

**Affiliations:** Department of Pharmacology, Institute of Pharmacology, Polish Academy of Sciences, Smętna Street 12, Kraków, 31-343 Poland

**Keywords:** Stress, Somatostatin, Medial habenula (MHb), Autoradiography, Pituitary

## Abstract

**Rationale:**

The involvement of somatostatin (SST) and its receptors in the pathophysiology of depression and stress has been evidenced by numerous studies.

**Objectives:**

The purpose of the present study was to find whether chronic mild stress (CMS), an animal model of depression, affects the SST receptors in the rat brain and pituitary, as well as the level of SST in plasma.

**Methods:**

In CMS model, rats were subjected to 2 weeks of stress and behaviorally characterized using the sucrose consumption test into differently reacting groups based on their response to stress, i.e., stress-reactive (anhedonic), stress-non-reactive (resilient), and invert-reactive rats (characterized by excessive sucrose intake). We measured specific binding of [^125^I]Tyr^3^-Octreotide, expression of mRNA encoding *sst2R* receptors in the rat brains, expression of SST and its receptors in rat pituitary, and the level of SST in the plasma.

**Results:**

The obtained results show decreases in binding of [^125^I]Tyr^3^-Octreotide in most of rat brain regions upon CMS and no significant differences between three stressed groups of animals, except for significant up-regulation of sst2 receptor in medial habenula (MHb) in the stress-reactive group. In the same group of animals, significant increase in plasma SST level was observed.

**Conclusions:**

There are two particularly sensitive sites distinguishing the response to stress in CMS model. In the brain, it is MHb, while on the periphery this predictor is SST level in plasma. These changes may broaden an understanding of the mechanisms involved in the stress response and point to the intriguing role of MHb.

## Introduction

Major depressive disorder (MDD) is a psychiatric illnes which touches about 5 % of the population. Despite numerous studies, the biochemical substrates of this disease (and thus new pharmacological treatment) are still not fully understood. Apart from classical neurotransmitters, some neuropeptides are also altered in certain brain regions in MDD. One of them is somatostatin (SST), widely distributed within the central nervous system (CNS), acting both as neurotransmitter and as neuromodulator (Engin and Treit 2009). Somatostatin binds with high affinity to five somatostatin receptors, SST1, SST2, SST3, SST4, and SST5, all belonging to the G-protein-coupled receptor family. All SSTRs share common signaling pathways such as the inhibition of adenylyl cyclase, activation of phosphotyrosine phosphatase (PTP), and modulation of mitogen-activated protein kinase (MAPK) via G-protein-dependent mechanisms (Patel 1999). Somatostatin is co-localized with gamma-aminobutyric acid (GABA) and is involved in regulating multiple aspects of physiological and behavioral stress responses (Engin et al. 2008; Lin and Sibille 2013).

It has been widely accepted that stress is one of the main causes of depression. Exposure to chronic stress is the leading risk factor associated with mood symptoms (Keller et al. 2007). The involvement of SST in the pathophysiology of depression and stress has been evidenced by numerous studies on human and animal models. In patients with MDD, the decreased SST level in the cerebrospinal fluid (CSF) was observed (Molchan et al. 1991; Frye et al. 2003). Low CSF SST level was significantly correlated with cortisol in depressive patients, suggesting the altered hypothalamic-pituitary-adrenal (HPA) axis function described in some depressed patients (Molchan et al. 1993; Holsboer 2001); however, the correlation between SST levels in CSF and plasma is not known. Intracerebral administration of SST to animals induces anxiolytic and antidepressant-like effects in the behavioral tests (Engin and Treit 2009). Moreover, the effects of SST2R and/or SST3R receptor agonists in the elevated plus-maze and forced swim tests were equivalent to effects of anxiolytic and antidepressant drugs, which indicates the potential role of these receptors subtypes in the antidepressant actions (Nilsson et al. 2012). Results obtained by Tripp et al. suggested an impaired excitation/inhibition balance in MDD potentially mediated by decreased GABA content; however, of relevance to this issue might be the results obtained recently, indicating a down-regulation of the SST levels in the anterior cingulate cortex, dorsolateral prefrontal cortex, and amygdala of MDD patients (Tripp et al. 2011; Sibille et al. 2011; Guilloux et al. 2012; Lin and Sibille 2015).

The purpose of the present study was to find whether chronic mild stress (CMS), an animal model of depression, alters the SST receptors in the rat brain and pituitary as well as the level of SST in the plasma. CMS is realistic and one of the best validated animal model of depression (Willner 2005; Wiborg 2013). In this model, rats were subjected to 2 weeks of daily stress and behaviorally characterized using the sucrose consumption test into differently reacting groups based on their response to stress, i.e., anhedonic (stress-reactive, stress-R), stress-resilient (stress-non-reactive, stress-NR), and invert reactive rats (characterized by excessive sucrose intake, stress-IR). The occurrence of resistance (or rather resilience) or susceptibility to stress in animals subjected to CMS has recently gained the interest of researchers (Bergström et al. 2008; Taliaz et al. 2011; Delgado y Palacios et al. 2011; Christensen et al. 2011; Żurawek et al. 2013; Faron-Górecka et al. 2014; Kolasa et al. 2014; Czéh et al. 2015), but the molecular signature underlying the observed differences in the behavioral responses to stress still remains unresolved. Since the previously obtained data suggested the role of SST in stress and depression, we decided to measure specific binding of [^125^I]Tyr^3^-Octreotide. Octreotide is SST2 and SST5 receptors (SST2R and SST5R, respectively) selective (Hannon et al. 2002; Siehler et al. 1998). Because the *sst5R* mRNA is present at low levels in the adult rodents brain (Hannon et al. 2002; Feuerbach et al. 2000), and the SST5R is mainly expressed in rat pituitary (Shimon 2003), we can say indirectly that brain receptor autoradiography using [^125^I]Tyr^3^-Octreotide allowed to observe mainly SST2R binding. Additionally, the expression of mRNA encoding *sst2R* receptors in the rat brains as well as expression of SST and its receptors in rat pituitary and the level of SST in the plasma of rats subjected to 2 weeks of CMS procedure were examined.

To the best of our knowledge, this is the first time when the correlation between stress and SST receptors in the brain sections was measured using this animal model of depression. Other studies were rather concentrated on the effect of antidepressant drugs on the SST levels using the elevated plus-maze and forced swim test (Pallis et al. 2009; Engin and Treit 2009) than on the impact of chronic stress. However, there are recent studies concerning the CMS model, but the authors focused their observation only on the hippocampus: the results obtained by Schaalan and Nassar (2011) demonstrate a possible antidepressant-like activity of octreotide in CMS due to its antioxidant/anti-inflammatory aptitude in the hippocampus, while Czéh et al. (2015) tried to determine which subset of GABAergic neurons are susceptible to the effect of chronic stress. They showed that chronic stress reduced by 15–25 % SST-ergic neurons in the CA1-2-3 areas of the hippocampus.

We report here that 2 weeks of chronic unpredictable stress significantly influenced the SST2R level in the various brain structures as well as the SST in the rat plasma. Moreover, it appears that one of the crucial structures involved in the response to stress, however often neglected in research, is the medial habenula nucleus (MHb).

## Materials and methods

The experiments were carried out in accordance with Bioethical Committee at the Institute of Pharmacology, Polish Academy of Sciences, Krakow, Poland.

### Animals

Male Wistar Han rats were purchased from Charles River, Germany. Animals’ weight was close to 300 g when adaptation for sucrose consumption was initiated and approximately 350 g at the start of stress procedure. Rats were brought to the laboratory 1 month prior to the start of the behavioral and biochemical experiments. Except when grouping was applied as a stress parameter, they were singly housed in plastic cages (40 × 25 × 15 cm) with food and water provided ad libitum, except when food or/and water deprivation was applied as a stress parameter. The standard 12-h light/dark cycle was only changed in course of stress regime.

### Behavioral testing

**Fig. 1:**
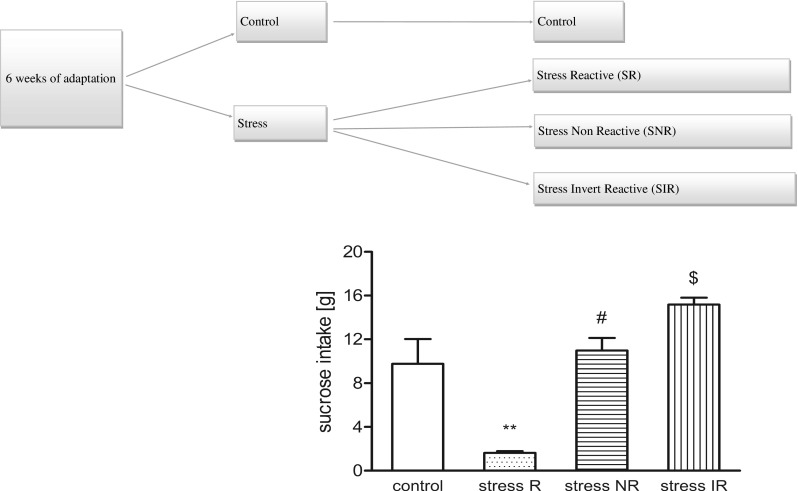
The scheme of 2 weeks of CMS procedure. In the final baseline test after 2 weeks of stress, sucrose intakes were significantly different between the controls and the stressed animals (*F*(1,40) = 21.11, *p* < 0.0001). The graph shows the differences in sucrose intake after 2 weeks of stress. The data represents mean ± S.E.M., *n* = 10 animals per group. For statistical analysis, a one-way ANOVA test was used with a Bonferroni post hoc test

Before the stress experiments, the animals were first trained to consume a tasty sucrose solution (1 %). The training procedure lasted for 6 weeks and consisted of 1-h testing sessions every week in which the sucrose solution was presented to the rats in their home cages after 14 h of food and water deprivation. Sucrose intake was measured after each drinking test as difference in bottle weight. Chronic mild stress experiments were performed according to the method described previously (Żurawek et al. 2015; Kolasa et al. 2014). Briefly, each week of the stress regime consisted of the following: two periods of food or water deprivation; two periods of 450 cage tilt; two periods of intermittent illumination (lights on and off every 2 h); two periods of soiled cage (250 ml water in sawdust bedding); two periods of paired housing; two periods of low intensity stroboscopic illumination (150 flashes/min); and two periods of no stress. All stressors were 10–14 h of duration and were applied individually and continuously, day and night. Animals were deprived of food and water for 14 h preceding each sucrose test, but otherwise food and water were freely available in the home cage. Control animals were remained undisturbed in a separate room with free access to food and water, except for a period of overnight deprivation for the sucrose consumption test. During the 2 weeks of stress, the sucrose consumption test was performed once a week, as described by Żurawek et al. (2015). The operational cutoff point between the control and stress-reactive group was based on arbitrary retrospective observations and was set at 7.5 g of sucrose consumption. Anhedonic animals (stress-reactive, stress-R) displayed decreased sucrose consumption to below 7.5 g when compared with the final baseline test. Animals resilient to stress (stress-non-reactive, stress-NR) characterized increased sucrose intake to above 7.5 g. The stress invert reactive (stress-IR) group was characterized by overstated level of sucrose intake over 13.5 g. As a results of the experiments, following groups of animals were selected: control rats (without stress procedure) and—as a result of 2 weeks of CMS: stress reactive/(stress-R); stress non-reactive/(stress-NR); stress invert reactive/(stress-IR). For biochemical studies, 10 animals were randomly selected within each group (Fig. 1).

### Somatostatin receptor autoradiography—binding of [^125^I]Tyr^3^-Octreotide

The rats were sacrificed by decapitation 24 h after the last sucrose test. The brains were rapidly removed and frozen using a heptane–dry ice mixture. Coronal brain sections (12 μm) were cut using a Jung CM 3000 cryostat microtome (Leica, Germany). The slices were thaw mounted on gelatine-covered microscope slides, air dried, and stored at −20 °C until use. In order to compare obtained slices with The Rat Brain Atlas (Paxinos and Watson 1998), the cresyl violet staining was performed. Receptor autoradiography was carried out as described by Ferone et al. (1999). As radioligand, the Somatostatin-28, Tyr^25^,[^125^I]-Leu^8^, D-Trp^22^ (Perkin Elmer, Germany) in 0.1 nM concentration was used. Nonspecific binding was determined by 1 μM non labeled rat somatostatin-14 (SST14, Prospec, Israel). Data were analyzed using one-way analysis of variance (ANOVA). A post hoc Bonferroni test was performed to locate differences between group means. The criterion for statistically significant differences for all experimental groups was set at *p* < 0.05. Data are expressed as percent changes in specific binding compared with the control group (expressed as 100 %) using the GraphPad Prism 5.0.

### In situ hybridization

The rat brain sections were fixed for 10 min in cold 4 % formaldehyde, briefly washed in PBS, and incubated for 10 min in an ice-cold acetic anhydride (0.25 %)–TEA (0.1 M) solution. The fixed brain slices were dehydrated in a graded series of alcohols and subjected to two 10-min incubations in chloroform to remove lipids. The prepared tissue slices were washed in ethanol and air dried. For in situ hybridization, the synthetic oligonucleotide complementary to *Rattus norvegicus sst2R* receptor (CGCGTTGCTTGTCATGTCGTAGTATGGCTC TGTCTGGTTG) was used. Oligonucleotide probes were labelled at the 3′ end with [^35^S]dATP (Hartmann Analytic, Germany) using terminal transferase (Fermentas, Lithuania). The probes were suspended to a final concentration of 1 × 10^6^ disintegration per minute (dpm) per 50 μl of hybridization buffer. The sections were hybridized with the labelled oligonucleotide for 18 h at 37 °C in a humidified incubator. Each slide was briefly washed twice in a 1× SSC solution at room temperature after hybridization, followed by four washes (15 min each) in 2× SSC with 50 % formamide at 42 °C and one for 15-min wash at room temperature in 1× SSC. The hybridized brain sections were rinsed with deionized water, dehydrated in increasing concentrations of ethanol, and air dried. Prepared tissue sections were placed into X-ray cassettes and exposed to film plates (Kodak) for 20 days at a temperature of −20 °C. The developed autoradiograms were analyzed, quantified, and normalized using Image Gauge software (Fujifilm, Japan).

### Gene expression of *sst* and its receptors in the pituitary

Using the qPCR method, we determined the mRNA levels of the *sst* and its receptors (*sst2R*, and *sst5R*) in the pituitaries of experimental animals from each group. The levels of the expressed genes were measured using a relative quantitative method (efficiency-corrected), with the peptidylprolyl isomerase A (*Ppia*) and ribosomal protein L32 (*Rpl32*) genes used as reference genes and the control group as a calibrator. RNA was reverse-transcribed with random hexamers to cDNA using a High Capacity cDNA Reverse Transcription Kit (Applied Biosystems, USA). Real-time PCRs were performed in a Chromo4 Real-Time Detector System (Bio-Rad Laboratories, Inc., USA) and Opticon Monitor Software v.3 (Bio-Rad, USA). The primer concentrations and annealing temperatures were checked and optimized experimentally. Real-time PCR amplification was performed in a total reaction volume of 20 μl, consisting of 10 μl Fast SYBR Green Master Mix (Applied Biosystems, USA), 0.5 μM forward primer, 0.5 μM reverse primer, and 5 μl cDNA template (ca. 5–10 ng reverse-transcribed total RNA per well). The sequences of the primers and the annealing temperatures for each target are given in Table 1. The thermal cycling profile consisted of an initial incubation at 95 °C for 10 s, followed by 40 cycles of denaturation at 95 °C for 15 s, annealing at a primer-specific temperature (Table 1) for 1 min, and ending with 1 cycle of final elongation at 72 °C for 7 min. For the qPCR reactions, cDNA was obtained from eight individuals from each group. The samples were run in duplicates, with no template controls in each experiment. A melting curve analysis was performed to confirm the amplification specificity of the PCR products. The results were calculated using qbasePLUS 2.0 software (Biogazelle) and ΔΔCt method (Hellemans et al. 2007). Reference genes stability was determined by calculating their M value (M) and their coefficient of variation on the normalized relative quantities (CV). For selected reference genes, the obtained average M = 0.680 and CV = 23.6 %, which are values accepted for heterogeneous samples (Hellemans et al. 2007; Kolasa et al. 2014).

**Table 1:** Primers used for qPCR amplifications and annealing temperatures used for each pairs of primers

Accession no.	Symbol	Forward primer (5′-3′)	Reverse primer (5′-3′)	*T*_ann_ [°C]
*NM_019348*	*Sst2R*	GCCATGGAGTTGACCTCTG	CATCTGCGATGGCCAGGTT	60
*NM_012882*	*Sst5R*	ATGGAGCCCCTCTCTCTGG	CGTCAGCCACGGCCAGGTT	63
*NM_012659*	*SST*	TTCTGCAGAAGTCTCTGGCG	ACAGGATGTGAATGTCTTCC	60
*NM_013226*	*Rpl32* ^a^	GTGAAGCCCAAGATCGTC	GAACACAAAACAGGCACAC	60
*NM_017101*	*Ppia* ^a^	TGACTTCACACGCCATAAT	AGATGCCAGGACCTGTATGC	60

^a^Reference genes used for normalization

### Peptide concentration in plasma

Upon decapitation, blood was collected into tubes containing EDTA and the plasma was then separated and stored at −80 °C for further analyses. The concentrations of the SST in the plasma were determined in duplicates by an enzyme-linked immunosorbent assays (ELISA) using commercially available kit for rat SST28 (Phoenix Pharmaceuticals, Germany). The ELISA procedures were performed as described by the manufacturers. Results were analyzed with the use of the standard curves.

## Results

### Behavioral experiments

In the final baseline test after 2 weeks of stress, the intake of 1 % sucrose solution was significantly lower in stress-reactive (stress-R) group vs. unstressed group (control) (1.71 ± 0.16 vs. 9.74 ± 2.16; *p* < 0.001). In the stress non-reactive animals (stress-NR), sucrose intake was comparable to the control group (11.31 ± 1.08 vs. 9.74 ± 2.16, respectively) in contrast to stress invert reactive group (stress-IR), where sucrose intake was significantly higher (15.62 ± 0.71 vs. 9.74 ± 2.16, respectively; *p* < 0.001). Figure 1 shows the sucrose intake of the rats used for the biochemical experiments.

### Binding of [^125^I]Tyr^3^-Octreotide in rat brains

**Fig. 2:**
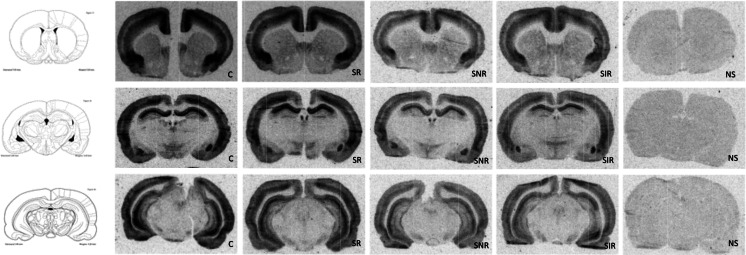
Representative autoradiograms of binding [^125^I]SST-28 in rat brains after 2 weeks of CMS procedure. *C*—control group, *SR*—stress-reactive group, *SNR*—stress non-reactive group, *SIR*—stress invert-reactive group, *NS*—non specific binding obtained using 1 μM SST14 non labeled rat somatostatin-14. Brain structures were selected using the Rat Brain Atlas Paxinos and Watson 1998

**Fig. 3:**
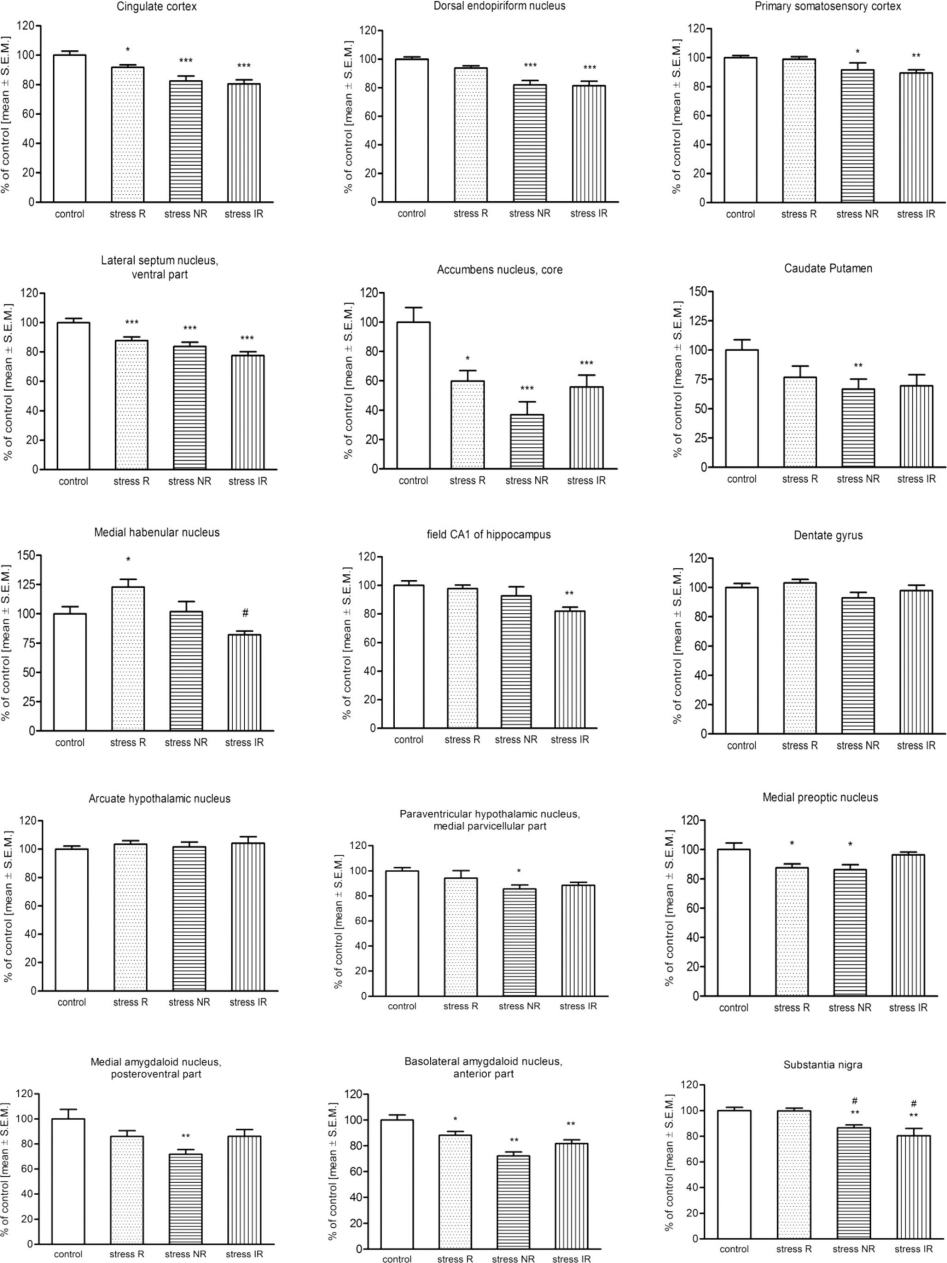
The percentage of specific binding of [^125^I]Tyr^3^-Octreotide in selected rat brain structures after 2 weeks of CMS procedure. The data represent mean ± S.E.M., *n* = 10 animals per group. For statistical analysis, a one-way ANOVA test was used with a Bonferroni post hoc test. *Asterisks* indicate statistical significance vs. control group; *p < 0.05, **p < 0.01, ***p < 0.001; #*p* < 0.01 statistical significance vs. stress reactive group (stress R)

The Somatostatin-28, Tyr^25^,[^125^I]-Leu^8^, D-Trp^22^ is SST analog that served as pharmaceutical octreotide acetate, which has a high affinity to SST2R and SST5R receptors (Patel 1999). In the control and all stressed groups, the high density of SST receptors using [^125^I]Tyr^3^-Octreotide binding in different brain areas was observed (Fig. 2). Rather a decrease of [^125^I]Tyr^3^-Octreotide binding in selected rat brain structures of limbic system was detected after 2 weeks of CMS procedure (Fig. 3), except for medial habenular nucleus (MHb), where in stress reactive group (stress-R) statistically significant increase of ligand binding was observed [*F*(3,33) = 7.502, *p* < 0.001, post hoc test *p* < 0.05 vs. control group]. On the other hand, we observed statistically significant decrease of [^125^I]Tyr^3^-Octreotide binding in this structure for stress invert reactive group (stress-IR), *p* < 0.01 vs. stress reactive group (stress-R). In the cingulate cortex (Cg) in all stressed groups, regardless of behavioral response, statistically significant decrease of [^125^I]Tyr^3^-Octreotide binding was observed [*F*(3,39) = 11.54, *p* < 0.0001]; however, this observation is more significant for stress-NR and stress-IR groups (*p* < 0.001 vs. control group). Similarly to changes observed in the Cg, also in lateral septum nucleus (LSV) [*F*(3,33) = 12.86, *p* < 0.0001], nucleus accumbens core (NAc) [*F*(3,37) = 10.25, *p* < 0.0001], caudate putamen (CPu) [*F*(3,37) = 4.54, *p* < 0.01], and basolateral amygdaloid nucleus, anterior part (BLA) [*F*(3,39) = 13.50], statistically significant decrease of [^125^I]Tyr^3^-Octreotide binding was observed. Interestingly, that decreased [^125^I]Tyr^3^-Octreotide binding was also observed in the dorsal endopiriform nucleus (DEn), primary somatosensory cortex (s1tr), and substantia nigra (SN) but only in stress-NR and stress-IR groups [*F*(3,39) = 14.71, *p* < 0.0001; *F*(3,35) = 5.27, *p* < 0.01; *F*(3,37) = 9.42, *p* < 0.0001, respectively]. Moreover, in the SN, the decreased of SST-28 binding was statistically significant vs. stress-R group (*p* < 0.01). In the medial amygdaloid nucleus posteroventral part (MePV) [*F*(3,35) = 4.55, *p* < 0.01] and paraventricular hypothalamic nucleus, medial parvicellular part (PaMP) [*F*(3,39) = 2.77, *p* < 0.05], the decrease of [^125^I]Tyr^3^-Octreotide binding, but only in stress-NR group, was observed. Interestingly, in the dentate gyrus (DG) of the hippocampus, we did not observe any statistically significant changes. In the CA1 field of the hippocampus, the statistically significant decrease was observed only in stress invert reactive (stress-IR, *p* < 0.01 vs. control). Conversely, in the medial preoptic nucleus (MPO) only in stress reactive (stress-R) and stress resilient (stress-NR) groups, the decrease of [^125^I]Tyr^3^-Octreotide binding was observed [*F*(3, 37) = 4.58, *p* < 0.01].

### Levels of mRNA encoding *sst2R*- in situ hybridization

**Fig. 4:**
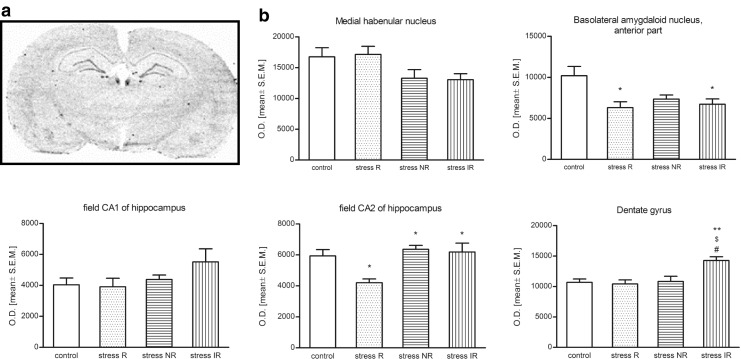
The level of mRNA encoding sst2R in rat brains after 2 weeks of CMS. **a** Representative autoradiogram of in situ hybridization of sst2R mRNA. **b** The level of sst2R mRNA in the rat brains. Data are expressed as a mean of optical density (*O.D*.; means ± S.E.M.; *n* = 10 animals per group). For statistical analysis, a one-way ANOVA test was used with a Bonferroni post hoc test. *p < 0.05, **p < 0.01 vs. control group, ^$^ *p* < 0.01 vs. stress R, ^#^ *p* < 0.01 vs. stress non-reactive (stress NR) group

The expression of mRNA encoding *sst2R* in rat brains after 2 weeks of CMS was observed in CA1 and CA2 fields of the hippocampus, DG, BLA, and MHb. In the MHb in stress-NR and stress-IR groups, the tendency to decrease in mRNA encoding *sst2R* was observed; however, this result did not reach statistical significance (Fig. 4). Conversely, in the field CA2 of the hippocampus, the increased s*st2R* mRNA expression in stress-NR and stress-IR was observed (*p* < 0.05 vs. stress R group). Moreover, in anhedonic (stress-R) animals, the statistically significant decrease of mRNA encoding *sst2R* was detected [*F*(3,30) = 0.008, *p* < 0.05 vs. control group). In the DG in stress-IR group, statistically significant increase of mRNA encoding *sst2R* was observed [*F*(3,39) = 0.0007, *p* < 0.01 vs. control, stress-R and stress-NR groups]. Only in BLA the decrease of mRNA encoding *sst2R* was observed in all stressed groups. This effect was statistically significant as compared to unstressed control group [*F*(3,25) = 0.001, *p* < 0.05].

### Gene expression in pituitary—RT-PCR

Using the qPCR method, we determined the mRNA levels of the *sst* and its receptors (*sst2R* and *sst5R*) in the pituitaries of experimental animals from each group. The levels of the expressed genes were measured using a relative quantitative method (efficiency-corrected), with the *Ppia* and *Rpl32* genes used as reference genes and “the control group” (mix of cDNA from all studied animals) as a calibrator. Because of technical reasons—plates for RT-PCR include 96 wells—we used eight samples per group to diminish inter run variations (8 samples per group × gene of interest + 2 reference genes × 4 groups). According to the results of a one-way ANOVA, there were significant differences in the expression of the *sst5R* [*F*(3,31) = 10.69, *p* < 0.0001]. We observed significant decreases in *sst5R* expression in all stressed groups as compared to the unstressed control. No changes were observed in the expression of mRNA of *sst* and the *sst2R* (Table 2).

**Table 2:** Expression of mRNA of *Sst* and its receptors in rat pituitaries. Data represents means ± S.E.M.; *n* = 8 animals per group. For statistical analysis, a one-way ANOVA test was used with a Bonferroni post hoc test

Target	Control	Stress R	Stress NR	Stress IR
*SST*	1.169 ± 0.309	1.513 ± 0.345	0.931 ± 0.147	1.167 ± 0.080
*Sst2R*	1.081 ± 0.190	1.068 ± 0.209	1.042 ± 0.133	1.336 ± 0.221
*Sst5R*	3.440 ± 0.736	1.803 ± 0.294 *	0.720 ± 0.101 **	0.622 ± 0.257 **

*p < 0.05; **p < 0.01, statistical significance vs. control group

### SST plasma level

**Fig. 5:**
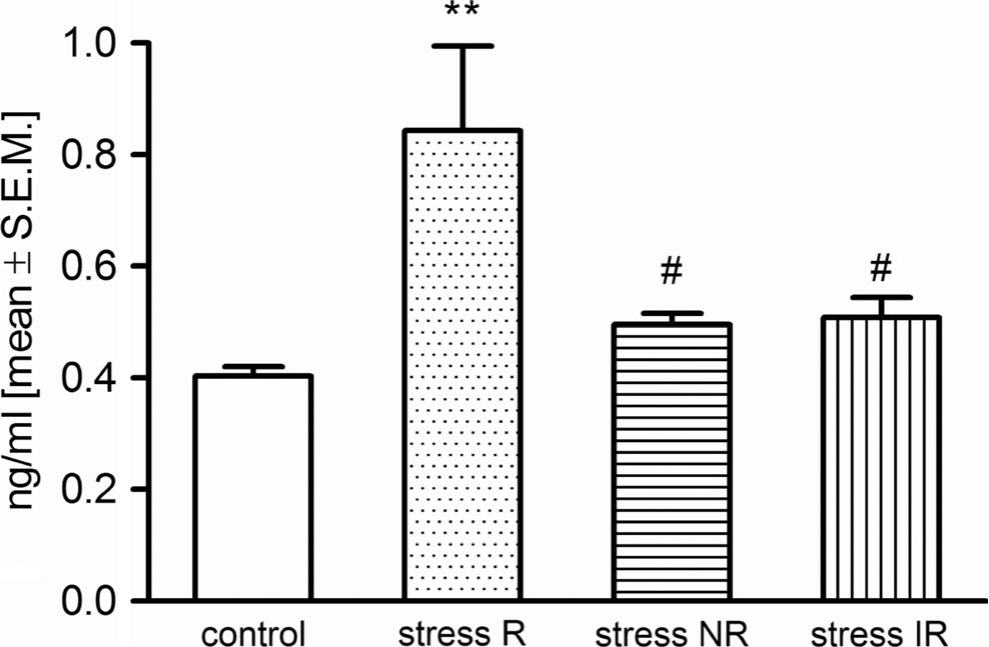
The plasma levels of SST in rats after 2 weeks of CMS procedure. Data represents means ± S.E.M.; *n* = 10 animals per group. For statistical analysis, a one-way ANOVA test was used with a Bonferroni post hoc test. *Asterisks* **p < 0.01 indicate statistical significance vs. control group; #*p* < 0.05 statistical significance vs. stress reactive group (stress R)

In the plasma, the level of SST was increased significantly in stress-R group after 2 weeks of CMS procedure (*p* < 0.05 vs. control group). Interestingly, in other stressed groups (NR and IR), the decreased SST level was observed as compared to stress-R group (*p* < 0.05) (Fig. 5).

## Discussion

### Chronic mild stress procedure

Full procedure of CMS (7 weeks of stress) is usually applied in research concerning the mechanisms of action of antidepressant drugs (Żurawek et al. 2015; Faron-Górecka et al. 2014); however, the symptoms of anhedonia can be observed after a shorter time of stress, as shown both in this study and by others (Kolasa et al. 2014; Luo et al. 2014; Luoni et al. 2014). Two weeks of stress led to the development of anhedonia manifested in stress-susceptible group of rats by the decreased sucrose consumption (stress-reactive, stress-R). Additionally, the group of animals not exhibiting any signs of anhedonia despite being stressed (stress non-reactive, stress-NR) and the group characterized by the increased sucrose consumption (stress invert-reactive group, stress-IR) were observed. Interestingly, despite the fact that the behavioral responses of stress-NR and stress-IR groups were different as shown by sucrose consumption, the molecular changes in these two groups were similar in present studies, although the results concerning the stress-IR group did not always reach statistical significance but they represent very similar trend as in stress-NR group. Similar correlation has been observed in our previous studies, where we have shown surprising similarity in gene expression and plasma ACTH levels in these two groups (Kolasa et al. 2014). These results might indicate parallelism underlying different ways to cope with stress conditions. Comparison of these two groups (stress-NR and stress-IR) showed that excessive intake of sucrose can be a compensation of anxiety state. Stress-IR group is also very interesting in the context of the phenomenon of the excessive eating under stress. The emotional eating under the influence of stress often leads to overeating and obesity (Singh 2014). Similar data have been already described by Dallman et al. (2003), who have shown that intake of palatable rewarding food reduced signs of stress, thereby showing the potential of “comfort eating” in stress relief.

### Impact of stress on the SST2R in rat brain

The results obtained using [^125^I]Tyr^3^-Octreotide suggest the potential role of SST2R and SST5R in stress-related behaviors. Octreotide is SST2R and SST5R selective (Hannon et al. 2002; Siehler et al. 1998). Because the *sst5R* mRNA is present at low levels in the adult rodents brain (Hannon et al. 2002; Feuerbach et al. 2000), and the SST5R is mainly expressed in rat pituitary (Shimon 2003), we can say indirectly that brain receptor autoradiography using [^125^I]Tyr^3^-Octreotide allowed to observe mainly SST2R binding. Such conclusion is also justified by studies using SST2R knockout mice, where the binding of this radioligand was not detected (Hannon et al. 2002). Our observations remain in agreement with available data, indicating the involvement of SST2R in anxiolytic and antidepressant actions. Infusion of a selective SST2R agonist reduced anxiety-like behavior in rats and also antidepressant-like effects were observed (Engin and Treit 2009). Using knockout mice, it has been shown that SST2R KO exposed to stress display a behavioral profile that is consistent with increased anxiety (Viollet et al. 2000). In our study, we observed, in many structures involved in the stress response, e.g., Cg or BLA, reduced SST2R binding in the groups of rats subjected to the stress procedures. The observed decrease of SST2R binding in BLA and LSV in all stressed groups might result as a response to the increased release of SST, since other studies reported the anxiolytic activity of SST in the amygdala and lateral septum. Noteworthy, the decrease of SST2R receptors binding has been observed in the SN in two stressed groups: stress-NR and stress-IR. Interestingly, we did not observe down-regulation of SST2R in stress-R group in this brain region, which may reflect the lack of compensatory mechanisms linked with neuroprotective effects of this neuropeptide. The decreased SST2R binding was observed in the present studies also in the NAc in all stressed groups. It remains in agreement with data obtained in studies indicating that SST is released neuronally in this brain region, and therefore, it can influence the level of its receptors (Pallis et al. 2006).

One has to be aware that activation of brain SST2R potently stimulates food intake and, independently, drinking behavior in rodents (Stengel et al. 2015). In our studies, prolonged stress induced down-regulation of SST2R in most brain regions studied—which may have influenced lower intake of sucrose solution; however, this effect was also observed in the stress-NR and stress-IR rats. Therefore, the stress itself seems responsible for down-regulation of SST2R binding. On the other hand, various stress conditions (i.e., exposure to nociceptive stimuli, immobilization, handling) led to increase in hypothalamic SST mRNA expression and peptide release (Arancibia et al. 1984, 2000), which suggests that the enhanced release of SST under stress conditions might be responsible for down-regulation of SST2R, as shown in our studies using [^125^I]Tyr^3^-Octreotide binding.

The most interesting results of our experiments is the binding of [^125^I]Tyr^3^-Octreotide in MHb. The habenula consists of set of nuclei, which regulate the release of multiple neuromodulators (Lecourtier and Kelly 2007). Most of studies concerning mood or anxiety disorders focused on lateral habenula nucleus (LHb) and/or did not distinguish the MHb from the LHb. The role of LHb in psychiatric disorders such as depression is well known (Hikosaka 2010; Aizawa et al. 2013; Christensen et al. 2013), while the role of MHb is neglected (Viswanath et al. 2014). Some studies have suggested that MHb plays a role in organizing or regulating many behaviors, including sleep (Cui et al. 2014), circadian rhythm (Zhao and Rusak 2005), pain (Shelton et al. 2012), fear responses (Agetsuma et al. 2010), and drug addiction (Velasquez et al. 2014). In humans and rats, lesions of this structure are correlated with schizophrenia (Lecourtier et al. 2004). Elevated activity in the MHb has also been linked with depression. In rat model of learned helplessness, increased activity in the MHb has been observed (Mirrione et al. 2014). Using zebrafish, it has been shown that MHb regulates the expression of fear (Mathuru and Jesuthasan 2013). Other data demonstrated that septo-habenular pathways regulate anxiety-related and fear responses (Yamaguchi et al. 2013). In the present studies, only in this structure we have observed an up-regulation of SST2R in stress-R group, while in other stressed groups no statistically significant changes (vs. unstressed control group) were observed. These results indicate that MHb is stress-sensitive, which makes this brain region an interesting site for stress resilience studies. Furthermore, the regulation of SST2R on the MHb level can serve as good potential site of action anxiolytic and antidepressant drugs. Data obtained by in situ hybridization indicate that changes in the binding of SST2R in anhedonic rats did not arise from changes in expression of this receptor (lack of the changes in *sst2R* mRNA expression). Also, other studies have shown that ferret exposure did not alter levels of *sst2R* mRNA within the MHb (Nanda et al. 2008).

As far as the hippocampus is concerned, despite the lack of SST2R binding in field of CA2 of the hippocampus, the changes of *sst2R* mRNA expression in this structure was observed. This effect can be associated with remodeling of neurons in the hippocampus. It has been shown recently that chronic unpredictable stress reduces the number of GABAergic interneurons (SST positive neurons) in the CA2 field of the hippocampus not only in anhedonic group but also in the one resilient to stress (Czéh et al. 2015). Additionally, only in stress-R group the decreased of *sst2R* mRNA expression was detected, in contrast to BLA, where in all stressed groups the decrease of *sst2R* expression has been observed. The changes in BLA are in opposite to the data obtained by Nanda et al., since they observed, in ferret predator paradigm, the increased *sst2R* mRNA expression (Nanda et al. 2008). This discrepancy may be due to a variety of stress. The ferret fear is rather severe and evolutionary conserved behavioral response essential for survival, while in our CMS paradigm, the mild stressors are introduced. Moreover, it has been shown that in rats different stress procedures can disrupt fear extinction (Shansky 2015). Recently, it has been demonstrated that amygdala activity was differentiated in high- and low-anxiety rats following midazolam treatment, which indicates the individual differences in local GABAergic activity (Skórzewska et al. 2015).

### Effect of stress on the pituitary

The pituitary, as a part of the hypothalamic-pituitary-adrenal (HPA) axis, plays a significant role in the stress response. Our previous studies have indicated that differential stress response in rats subjected to 2 weeks of CMS was accompanied by changes in CRH-family gene expression at the pituitary level (Kolasa et al. 2014). It has been demonstrated that *sst2R* and *sst5R* mRNA are expressed in the pituitary gland in the rats (Zhang et al. 1999). Therefore, we decided to measure *sst*, *sst2R*, and *sst5R* gene expression in pituitaries of the same rats after CMS procedure. In all stressed groups of animals, the decreased *sst5R* expression was observed, which indicates the role of this type of receptor in stress responses, although it did not differentiate the reaction to CMS, since the alterations in sst5R mRNA expression were the same in stress-R, stress-NR, as well as stress-IR rats. This receptor has been shown to be involved in GH-secreting adenomas and prolactinomas (Moore et al. 2005); however, its link with the stress response in rat pituitary was not often suggested.

### SST plasma level

Contrasting to the central effects, SST actions in the periphery are largely inhibitory (Stengel and Taché 2013). SST is released from nerve endings, neuroendocrine, inflammatory, and immune cells in response to a wide range of inflammatory mediators (Patel 1999). Our data indicated that changes of SST level in the periphery are correlated with behavioral response. In the stress-R group, the increased SST level was observed in plasma. Interestingly, the present studies showed that in other stressed groups (i.e., stress-NR and stress-IR), SST levels were not different from the unstressed control group. This observation indicates that the regulation of peripheral SST levels can be a predictor of sensitivity to stress.

## Conclusion

The data obtained in the present studies confirm the involvement of SST and its receptors (SST2R and SST5R) in response to stress. It seems that there are two particularly sensitive sites distinguishing the response to stress in CMS model. In the brain, it is MHb and its sensitive reaction at the level of SST2R, while on the periphery this predictor is SST level in plasma. These changes may broaden the understanding of the mechanisms involved in the stress response and point to the intriguing role of MHb; however, further studies focusing on this brain region are required.
